# Configurational pathways to pediatric e-bike injury severity: an fsQCA study of global evidence

**DOI:** 10.3389/fpubh.2025.1738100

**Published:** 2026-01-06

**Authors:** Yanni Li, Qing Li, Mingming Liang

**Affiliations:** 1 Tuberculosis Prevention and Control Institute, Anhui Provincial Center for Disease Control and Prevention, Hefei, Anhui, China; 2 School of Health Management, Anhui Medical University, Hefei, Anhui, China; 3 Department of Obstetrics and Gynecology, Anqing Municipal Hospital, Anqing, Anhui, China

**Keywords:** adolescent trauma, e-bike, fsQCA, injury severity, pediatric injury, risk behavior, traffic safety

## Abstract

**Background:**

E-bike use among children and adolescents has surged worldwide, raising concerns about injury severity and safety management. While prior research has identified multiple risk factors, the complex interplay among study and contextual characteristics influencing injury outcomes remains poorly understood.

**Objective:**

This study seeks to uncover the configurational pathways that contribute to increased severity in pediatric e-bike injuries. It employs fuzzy-set qualitative comparative analysis (fsQCA) to integrate both methodological and contextual factors drawn from global evidence.

**Methods:**

Twenty-two case was calibrated across seven conditions: research quality, study design, use of quantitative severity indicators, head injury focus, multisystem injury perspective, child or adolescent sample, and inclusion of risk behavior variables. The fsQCA was applied to explore necessary conditions and sufficient configurations explaining the presence or absence of severe injury outcomes. Cases were calibrated into fuzzy sets based on the presence or absence of these conditions, and the algorithm was employed to identify parsimonious and intermediate solutions for both the presence and absence of severe injury outcomes.

**Results:**

The necessity analysis revealed that high-quality research, robust study design, and child-focused samples were common among cases reporting significant injury severity differences. The configurational analysis revealed three sufficient pathways leading to increased pediatric e-bike injury severity. Across these configurations, the absence of behavioral risk analysis combined with rigorous design consistently contributed to low-severity outcomes, while multi-system perspectives and pediatric-focused samples played complementary roles. Robustness analyses confirmed the stability of the identified causal structures across analytical thresholds.

**Conclusion:**

Pediatric e-bike injury severity is shaped by multidimensional interactions among methodological rigor, injury focus, and population characteristics rather than single risk factors. The fsQCA approach provides a novel analytical framework for disentangling heterogeneous evidence and offers new insights into designing effective injury prevention and safety policies for children.

## 1 Introduction

An electric bicycles (e-bikes) is commonly defined as a bicycle equipped with an electric motor that assists pedaling or provides independent propulsion. The rapid global adoption of e-bike has fundamentally reshaped patterns of personal mobility. Once viewed primarily as a recreational device, the e-bike has become a popular mode of short-distance transportation in both developed and developing countries (1). While this shift offers environmental and economic benefits, it also raises growing concerns regarding traffic safety, particularly for children and adolescents (2). Compared with traditional bicycles, e-bikes can reach higher speeds with less physical effort, increasing both exposure to road risks and the likelihood of more severe injuries when crashes occur (3).

Children are widely recognized as vulnerable to motorized recreational injuries. The growing popularity of e-bikes further heightens this risk. Depending on their operational mode, e-bikes are generally categorized into pedal-assist and throttle-controlled types (4). Pedal-assist e-bikes supply power only when the rider is pedaling and usually have a speed limit (20 mph in the United States and 25 km/h in China and Europe), after which the motor stops providing assistance. In contrast, throttle-controlled e-bikes can operate solely through the throttle without pedaling, and often reach higher speeds. Variations in power and design across these types directly influence potential speed and handling difficulty, which is particularly critical for child users (5).

Emergency department data from different regions show that both the number and severity of e-bike injuries among children have increased rapidly in recent years (6). In high-income countries, the frequency of such injuries is now similar to that of motorcycle-related cases. In many low- and middle-income countries, the lack of safe infrastructure and weak law enforcement make the situation even worse (7).

Findings on the specific determinants and mechanisms of the severity of e-bike injuries among children remain inconsistent. Most studies emphasize describing trends and patterns in the scale of injuries. Previous research has identified several key risk factors, including not wearing a helmet, insufficient protective gear, and dangerous riding behaviors (7, 8). However, existing studies rely on linear statistical models that treat these factors as independent variables. These approaches often overlook how behavioral, technical, and environmental elements interact with each other in real-world settings. Therefore, inconsistent results have not been obtained in discussions of the severity of injuries sustained by e-bike riders. The integrated or configurational pathways leading to serious injuries among young e-bike riders have not been systematically investigated.

From a public health perspective, it is more meaningful to understand how different conditions work together to cause severe injuries in children than to look only at the separate effects of individual factors (4). For example, the type of vehicle, the road environment, and the level of caregiver supervision may interact to shape injury severity in ways that single-variable analyses cannot reveal (9). Such relationships are often configurational rather than simply additive, meaning that several different combinations of factors can produce similar outcomes, and the same factor may have different effects in different contexts (10). To address this gap, the present study applies fuzzy-set qualitative comparative analysis (fsQCA) to identify multiple, coexisting causal pathways associated with pediatric e-bike injury severity. By synthesizing international empirical evidence, this study aims to explain how different contextual and methodological factors jointly influence the severity of injury outcomes. The findings provide a nuanced understanding of risk formation, offering guidance for policymakers, healthcare professionals, and educators seeking to enhance child safety within the rapidly evolving landscape of electric mobility.

## 2 Study theory

Despite the rapid growth of pediatric e-bike use globally, research on injury mechanisms has remained fragmented, often focusing on isolated factors such as helmet use, age, speed, or roadway characteristics. However, emerging injury science highlights that pediatric traffic injuries rarely result from a single determinant; instead, they arise from complex interactions among individual, behavioral, environmental, and systemic factors. This understanding aligns with complex causality theory, which emphasizes conjunctural causation, equifinality, and asymmetric relationships among conditions leading to an outcome. Growing evidence shows that pediatric mobility injuries are shaped by multiple interacting factors rather than single independent risks. This aligns with complex causality theory, which emphasizes conjunctural and asymmetric causal patterns that jointly produce injury outcomes (11, 12). Within this framework, injury severity is understood as the result of specific configurations of individual characteristics, behavioral factors, environmental conditions, and study-level attributes. Such a perspective supports the use of fsQCA, a method designed to identify multi-factorial causal pathways rather than isolated predictors.

From a broader systems perspective, public health systems theory provides an additional theoretical foundation for a configurational approach. Pediatric injury severity reflects the functioning of interconnected subsystems—including clinical reporting, surveillance quality, community safety practices, and policy environments. Variations in study design and measurement across global research contexts are not merely methodological artifacts. They reflect underlying system capacities and information flows within public health infrastructures. Thus, analyzing how research quality and measurement choices combine with population-level factors can help reveal systemic gaps in injury monitoring and the structural determinants that influence the detection and classification of severe injuries. At the systems level, public health systems theory highlights that variations in injury severity reporting reflect differences in surveillance capacity, data quality, clinical pathways, and policy context (13, 14). Differences in study design and measurement in global pediatric e-bike injury research therefore represent not only methodological heterogeneity but also underlying system-level constraints that shape what risks are captured and how severity is classified.

The safety ecology model further conceptualizes injuries as emerging from interactions between the child, the vehicle, and social-physical environments (15, 16). In the case of e-bikes, ecological factors are often inconsistently measured, leading to heterogeneous evidence. Interpreting “research design—population—behavioral variables” as configurational components of this ecological system provides theoretical grounding for fsQCA.

Taken together, these theoretical perspectives justify a configurational approach for analyzing pediatric e-bike injury severity. By integrating complex causality, public health systems dynamics, and ecological safety interactions, fsQCA allows us to identify how specific combinations of study design quality, population characteristics, and behavioral indicators jointly contribute to high- or low-severity outcomes across global research contexts.

## 3 Materials and methods

### 3.1 Study design and analytical framework

This study employed fuzzy-set qualitative comparative analysis (fsQCA) to explore configurational pathways leading to severe pediatric e-bike injuries. FsQCA offers a case-oriented analytic logic that integrates qualitative reasoning with quantitative rigor (17). Unlike conventional regression models focusing on net effects, fsQCA identifies combinations of conditions sufficient or necessary for a given outcome. Each published empirical study was treated as a single analytical case characterized by specific features, including research design, injury type, and contextual attributes. This approach enabled systematic cross-case comparison to uncover how different combinations of conditions explain varying conclusions about e-bike injury severity in children.

### 3.2 Literature search and case selection

A systematic search was performed in PubMed, Web of Science, Embase, and Google Scholar without time restrictions up to July 2025. Search terms combined controlled vocabulary and free-text expressions: (“electric bicycle*” OR “e-bike*” OR “electric bike*” OR “pedelec*”) AND (“child*” OR “pediatric” OR “paediatric” OR “adolescent*” OR “youth”) AND (“injur*” OR “trauma” OR “accident*” OR “crash*” OR “collision*”). Reference lists and citation networks were manually screened to ensure comprehensive coverage.

Studies were eligible if they (1) included participants aged 0–18 years involved in e-bike-related injuries; (2) provided original empirical data with quantitative or mixed-method designs; and (3) reported measures of injury severity, mechanisms, or associated risk factors. Excluded were studies focusing exclusively on adults, those limited to conventional bicycles, and publications lacking primary data or adequate methodological detail. Following PRISMA standards, studies met the inclusion criteria and were retained as analytical cases for fsQCA.

### 3.3 Calibration of fuzzy-set membership

For each case, fuzzy-set membership scores were assigned on a continuum from 0.0 (complete non-membership) to 1.0 (complete membership), reflecting the extent to which a condition or outcome was present. Calibration followed established guidelines (18), applying three qualitative anchors: full membership (1.0), crossover (0.5), and full non-membership (0.0). Intermediate scores (0.33, 0.67) were used when evidence partially supported a condition.

### 3.4 Outcome variable and condition variables

#### 3.4.1 High severity of pediatric e-bike injury (Y)

The outcome represented whether a given study concluded that e-bike-related injuries among children were more severe than those from conventional bicycles or comparable non-motorized vehicles. Studies reporting increased rates of hospitalization, head or facial trauma, or multi-system injuries were coded as exhibiting high-severity outcomes. The outcome and condition variables were calibrated according to the principles of fuzzy-set qualitative comparative analysis, with membership scores ranging from 0 (complete non-membership) to 1 (full membership). Studies reporting higher hospitalization rates, head or facial trauma, or multisystem injuries were coded as exhibiting high-severity outcomes. Full membership (1.0) was assigned when comparative or statistical evidence clearly indicated greater severity. Partial membership (0.5) was assigned when severity differences were described qualitatively without formal testing. Studies reporting no difference or mixed findings were coded as 0.0.

Seven condition variables were included to capture both methodological rigor and thematic focus. Calibration decisions were informed by the conceptual correspondence between each condition variable and clinical or public health theory. High research quality was evaluated using adapted critical appraisal criteria encompassing study design, sample representativeness, confounder control, and analytical robustness (1.0 = high; 0.67 = moderate; 0.33 = low; 0.0 = poor). Cohort or case–control design distinguished longitudinal or comparative designs (1.0) from cross-sectional studies (0.0). Quantitative severity indicators assessed the use of standardized injury metrics such as the injury severity score or Abbreviated Injury Scale (1.0 = clearly used; 0.5 = partially used; 0.0 = not used). Head/facial injury focus captured studies emphasizing cranial or craniofacial trauma (1.0 = specific analysis; 0.67 = descriptive mention; 0.0 = not addressed), while multisystem or lower extremity injury focus identified inclusion of polytrauma or limb injuries (1.0 = present; 0.0 = absent). Exclusively pediatric population differentiated child- and adolescent-focused samples (1.0) from mixed-age designs (0.5) and adult-only samples (0.0). Finally, behavioral and environmental risk factor analysis reflected whether a study examined helmet use, traffic exposure, or environmental hazards (1.0 = included; 0.0 = not included). In examining behavioral conditions across the included studies, we applied a dual coding criterion. First, we required the explicit reporting of risk-related behaviors. Second, we assessed whether the study design had the methodological capacity to capture such behaviors. This approach minimizes the chance that missing behavioral information will be mistakenly treated as evidence of genuinely low-risk behavior. A standardized calibration table is shown in Supplementary Table S1. Calibration was independently performed by two researchers, and any inconsistencies were resolved through discussion and consensus to ensure reliability and transparency.

All fsQCA analyses were conducted in R (version 4.1.0) using the QCA package. The procedure comprised four integrated steps. First, a necessity analysis was performed to test whether individual conditions were essential for the outcome, adopting a consistency threshold of 0.90. Second, a truth table was constructed to represent all 2^7^ = 128 logically possible configurations of the seven causal conditions, with only those meeting minimum empirical (≥1 case) and consistency (≥0.80) thresholds retained as outcome-producing pathways. Third, Boolean minimization using the Quine–McCluskey algorithm generated complex, parsimonious, and intermediate solutions, among which the intermediate solution was prioritized for its balance between empirical adequacy and theoretical plausibility. Finally, solution interpretation identified the causal configurations leading to high injury severity, distinguishing between core and peripheral conditions based on their presence across solution types.

### 3.5 Robustness and sensitivity analysis

To evaluate the stability of the fsQCA findings, robustness and sensitivity checks were performed by systematically varying key analytical parameters, including consistency thresholds for truth table coding (0.75, 0.80, 0.85), frequency thresholds (1 or 2 cases), and proportional reduction in inconsistency (PRI) thresholds (0.50, 0.65) to account for potential simultaneity effects. Configurations were deemed robust if they consistently appeared across these alternative specifications. In addition, the analysis included the negation of the outcome (~Y), representing the absence of high-severity injury conclusions, to explore asymmetric causality.

### 3.6 Ethical considerations

This study synthesized data exclusively from previously published research and did not involve primary data collection from human participants. As a secondary analysis of publicly available literature, it was exempt from institutional review board approval.

## 4 Results

### 4.1 Study characteristics and analytical scope

**Figure 1:**
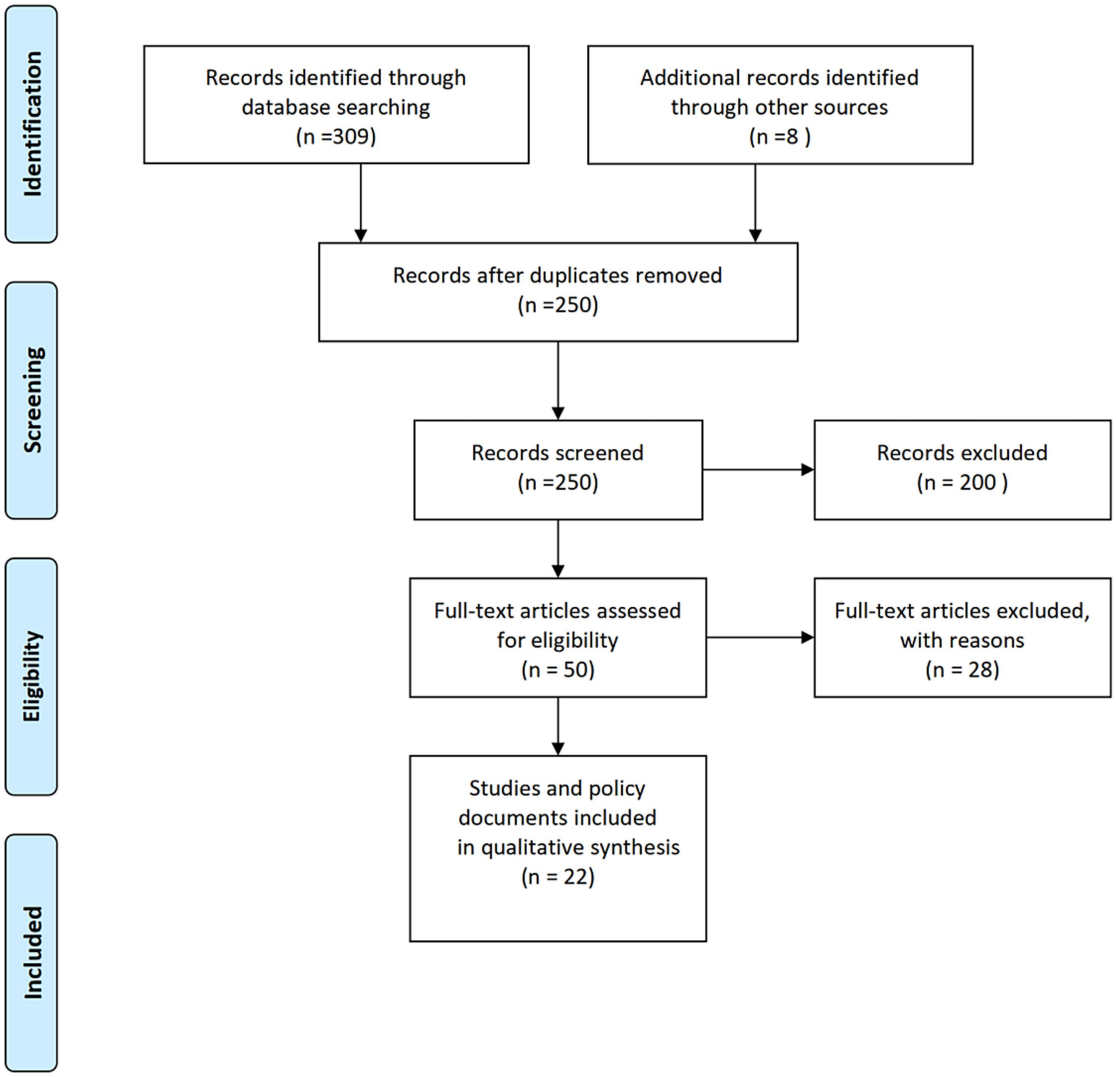
PRISMA flow diagram for study selection on pediatric electric vehicle injuries.

This study synthesized evidence from 22 peer-reviewed publications (Supplementary Table S2) examining pediatric injuries associated with electric bicycles, published between 2015 and 2025 (Figure 1) (1–3, 5, 19–36). The included studies represented diverse geographic contexts, spanning the United States, China, Israel, Australia, and multiple European nations. Most focused on the clinical patterns, mechanisms, and determinants of injury among children and adolescents aged 0–18 years. Within the fsQCA framework, each publication was treated as an individual case defined by its methodological characteristics, study population, and analytical emphasis.

### 4.2 Comparative patterns, mechanisms, and risk factors

Comparative analyses across micromobility device types consistently show that electric propulsion substantially increases both injury incidence and severity among pediatric users. Studies utilizing NEISS datasets and hospital-based cohorts report higher rates of severe trauma, hospitalization, and craniofacial injury in e-bike or e-scooter users compared with pedal cyclists (3, 5, 19, 21, 29). Device-specific factors, such as higher speed and mechanical instability, contribute to these differences. These differences are largely attributed to higher speed and mechanical instability, although contextual determinants remain insufficiently explored (35, 37).

Adolescents aged 12–18 years represent the highest-risk group, reflecting developmental tendencies toward sensation-seeking, limited traffic experience, and inconsistent helmet use (2, 22, 36). A male predominance and seasonal peaks during summer or after-school hours are also observed (23, 34, 38). Although injuries among younger children occur less frequently, their physiological fragility predisposes them to more severe consequences.

Clinically, the head and extremities are the most affected body regions. Head trauma ranges from superficial abrasions to severe brain injuries, frequently associated with the absence of helmet protection (1, 3). Fractures and lacerations of the limbs are common. Trauma to the trunk and face, while less common, can result in long-term disability (1, 9, 28). The predominant mechanisms include falls and collisions (Supplementary Figure S1), which was developed by the authors based on a synthesis of existing theoretical frameworks in injury epidemiology, including the safety ecology model, complex causality theory, and public health systems theory. The model integrates evidence from the included studies to illustrate how individual, behavioral, environmental, and system-level factors interact to influence injury severity. Compared with conventional bicycles, e-bike crashes result in higher rates of internal organ injury and longer hospital stays (20, 25, 31).

Based on the comprehensive evidence from the included studies and referring to previous literature, it was found that the risk factors for children’s electric bicycle injuries involve user, vehicle and environmental factors (Supplementary Figure S2). The framework summarizes the major behavioral, environmental, and study-design-related factors identified in the literature and depicts their potential interactions in the fsQCA model. Immature motor and cognitive capacities, coupled with inexperience, distraction, and inadequate protective equipment, increase vulnerability (24, 27, 30). Vehicle-related contributors include excessive speed, design instability, and poor maintenance (3, 29, 32), while environmental hazards such as uneven roads, dense traffic, and insufficient parental supervision further elevate risks (34, 38, 39).

### 4.3 Necessity analysis for severity outcomes

The necessity analysis examined whether any single condition consistently functioned as a prerequisite for the absence of high-severity outcomes (~Y). As presented in Table 1, two methodological conditions demonstrated notably high necessity: B_Design (cohort/case–control study design) and A_Quality (high research quality), with consistency values exceeding 0.90, indicating that rigorous methodological architecture is crucial for reliably identifying low-severity injury patterns in pediatric e-bike research. Consistency thresholds were set at 0.90 for necessity analysis, in line with standard fsQCA practice, ensuring that only conditions almost always present in low-severity outcomes were identified as necessary. Coverage values were reported to indicate the proportion of cases explained by each condition.

**Table 1:** Necessity analysis results (negative outcome: ~Y).

Condition	Consistency (inclN)	Coverage (RoN)	Raw coverage (covN)
A_Quality	0.956	0.473	0.487
B_Design	1.000	0.438	0.488
C_SeverityIndicator	0.413	0.610	0.301
D_HeadInjuryFocus	0.522	0.407	0.273
E_MultiSystem	0.434	0.669	0.350
F_ChildSample	0.956	0.238	0.396
G_RiskBehavior	0.435	0.857	0.557

The configurational results (Table 2) revealed that high-quality and well-designed studies focusing on head injury and severity indicators among pediatric samples consistently led to higher risk identification. Configurations lacking behavioral variables but emphasizing methodological rigor and pediatric focus emerged as dominant pathways to high-risk outcomes. In contrast, multi-system perspectives served as peripheral enhancers rather than core determinants. These findings underscore that the salience of risk identification in e-bike injury research depends more on methodological robustness and population focus than on the inclusion of behavioral or systemic variables.

**Table 2:** Configurations leading to high severity of pediatric e-bike injury.

Condition	Configuration 1	Configuration 2	Configuration 3	Configuration 4	Configuration 5
High research quality	●	●	●	⊗	●
Cohort/case–control design	●	●	●	●	●
Quantitative severity indicator	●	●	⊗	⊗	⊗
Head/facial injury focus	●	●	⊗	⊗	●
Multisystem/lower limb injury	⊗	●	⊗	⊗	●
Exclusively pediatric sample	●	●	●	●	●
Risk behavior analysis	⊗	⊗	⊗	⊗	⊗
Number of cases (*n*)	1	10	2	1	1
Raw consistency	1.000	0.940	0.932	0.906	0.865
PRI	1.000	0.922	0.801	0.000	0.601

“●” indicates presence of the condition (core condition if appearing in parsimonious solution); “⊗” indicates absence of the condition; blank spaces indicate “do not care” conditions.

### 4.4 Configurational pathways to severity outcomes

The fsQCA identified three distinct sufficient pathways associated with reduced pediatric e-bike injury severity (Table 3). Consistency and coverage thresholds were applied to ensure robust interpretation. Configurations with consistency ≥0.8 were considered sufficiently reliable to produce the outcome. Raw coverage values indicate the proportion of all low-severity cases explained by each configuration, while unique coverage represents the proportion uniquely accounted for by that pathway.

**Table 3:** Conservative solution for the negative outcome (~Y).

Configuration	Consistency (inclS)	PRI	Raw coverage (covS)	Unique coverage (covU)
Configuration 1: A_Quality * B_Design * C_SeverityIndicator * D_HeadInjuryFocus * F_ChildSample *~G_RiskBehavior	0.946	0.932	0.420	0.047
Configuration 2: A_Quality * B_Design * D_HeadInjuryFocus * E_MultiSystem * F_ChildSample *~G_RiskBehavior	0.896	0.861	0.408	0.035
Configuration 3: B_Design *~C_SeverityIndicator *~D_HeadInjuryFocus *~E_MultiSystem * F_ChildSample *~G_RiskBehavior	0.777	0.504	0.163	0.140

“~” denotes the absence or negation of the condition.

Configuration 1 describes studies characterized by high methodological quality, standardized severity measures, and a clear focus on head injuries in pediatric cohorts, without behavioral risk analysis. Such studies frequently evaluate helmet effectiveness and structured supervision, reflecting contexts where child-specific protective practices effectively mitigate severe outcomes. These findings suggest that systematic assessment of head protection using validated metrics enhances detection of high-severity patterns.

Configuration 2 highlights the synergistic role of multisystem analysis (physical, biomechanical, and environmental safety factors) combined with head injury focus and high-quality study design. The configuration demonstrates that comprehensive, multi-domain safety evaluation frameworks can effectively identify conditions under which severe pediatric e-bike injuries are preventable. Studies within this pathway often examine interactions between helmet use, vehicle design features, and environmental infrastructure, revealing how integrated interventions reduce injury severity.

Configuration 3 describes studies with strong design quality focusing on pediatric populations but lacking extensive severity indicators, head injury specificity, or multisystem analysis. Despite these methodological simplifications, the absence of behavioral risk factor analysis still associates with severe outcomes. This configuration suggests that injuries were more severe in children due to design flaws remains valid even if the study did not employ specific severity indices or focus on particular injury types, thereby strengthening the causal chain linking design deficiencies to serious pediatric trauma.

### 4.5 Core and peripheral conditions

Across all three pathways, two conditions emerged as core elements present in both parsimonious and intermediate solutions: B_Design (rigorous study design) and ~G_RiskBehavior (absence of behavioral risk analysis). This dual core indicates that methodological robustness combined with contexts where risk behaviors are either absent or not analytically emphasized constitutes the foundational architecture for identifying severity patterns. F_ChildSample (exclusively pediatric focus) appeared as a peripheral condition in most configurations, suggesting that age-appropriate sampling enhances but does not independently determine severity outcomes.

The robustness assessments provide strong evidence for the stability of the fsQCA model. The persistence of key pathways under varying analytical thresholds supports the validity of the identified causal mechanisms and reinforces confidence in the configurational interpretation of pediatric e-bike injury outcomes.

## 5 Discussion

This fsQCA study systematically examined global evidence on pediatric injuries associated with electric bicycles and other powered micromobility devices, identifying three distinct configurational pathways leading to injury severity outcomes. These findings collectively highlight that severity outcomes emerge not from single determinants but from interdependent, context-sensitive configurations of methodological, clinical, and behavioral conditions. The configurational results indicate that the severity of pediatric e-bike injuries is shaped by a set of recurring structural patterns rather than any single dominant factor. Across studies with varying levels of measurement detail, injury-type specificity, and analytical breadth, children consistently appear more vulnerable to severe harm when exposed to design flaws, inadequate head protection, and limited supervisory safeguards. High-quality studies tend to capture the protective value of validated severity assessment and helmet use more clearly, while multi-domain evaluations reveal how vehicle structure, rider protection, and environmental conditions interact to moderate severe outcomes. Even in studies with simplified measurement frameworks, design-related hazards remain strongly linked to greater injury severity, underscoring the amplifying effect of structural risks in younger riders. The identification of multiple equifinal pathways challenges conventional variable-oriented assumptions that prioritize isolated risk factors.

The findings of this study are consistent with recent research demonstrating that electric micromobility devices pose substantially greater injury risks for children and adolescents compared with traditional bicycles (5, 40, 41). These elevated risks can be attributed to several interrelated mechanical and behavioral factors. From a mechanical standpoint, e-bikes and e-scooters typically reach higher maximum speeds, possess greater overall mass, and have smaller wheelbases and narrower tires, which collectively reduce maneuverability and increase the kinetic energy transferred upon impact. Such characteristics heighten the probability of control loss, particularly on uneven surfaces or in congested urban settings, resulting in more frequent and severe injuries. From a behavioral and developmental perspective, younger riders tend to have underdeveloped motor coordination, limited risk perception, and a tendency toward sensation-seeking behaviors, which may increase unsafe riding practices such as speeding, riding on roadways shared with motor vehicles, or neglecting traffic rules (19, 20, 39). Compounding these vulnerabilities is the inconsistent or incorrect use of protective equipment—especially helmets—which has been repeatedly identified as a critical determinant of head and facial injury severity (8, 24). Furthermore, the rapid proliferation of e-micromobility devices has often outpaced the establishment of age-appropriate regulations and infrastructure adaptations, leaving younger users exposed to risks in environments originally designed for adult cyclists or motor vehicles.

Earlier studies often examined single factors, like whether riders wore helmets, their age, or the type of roads they used. These studies produced mixed results and did not explain how these elements combine to affect injury severity. In contrast, our fsQCA analysis demonstrates that when strong research design, good data quality, and behavioral control occur together, the risk of serious injury can be identified even in different environments. This suggests that the way studies are structured and the contexts they capture are as important as the medical or behavioral factors themselves. Previous studies demonstrated that analytical completeness, rather than technical sophistication alone, determines study robustness. The present analysis contributes to this discussion by specifying how certain combinations of methodological and contextual deficiencies jointly predict negative outcomes. Moreover, the observed interaction between child sample and risk behavior reflects recent literature on vulnerable road users, suggesting that child-related studies demand behavioral modeling frameworks that account for environmental and cognitive variability (41).

The configuration study highlighting multisystem integration as a pathway to lower injury severity aligns with established public health frameworks advocating for coordinated efforts among engineering, behavioral education, and regulatory domains. Evidence suggests that interventions integrating multiple perspectives are more effective in reducing severe injuries and enhancing overall prevention outcomes. These findings underscore the importance of adopting a systems-based approach to pediatric e-bike safety, emphasizing the interplay between individual behavior, technological design, and policy enforcement.

The configurational results have clear implications for injury prevention and child safety. The findings show that good research design and reliable data are important for accurate risk assessment. Building a standard injury reporting system that links medical, behavioral, and environmental information can help improve both research and policy decisions (22). The results also highlight the need for coordinated safety actions. Education on safe riding, helmet use, traffic rules, and better road design should be combined to reduce risks (2, 32). In addition, studies with low injury severity often involve controlled environments, showing that supervision and safe riding areas can help protect young riders. Together, these results suggest a practical, multi-level strategy that joins education, regulation, and infrastructure improvement to make e-bike use safer for children and adolescents.

The findings of this study carry important implications for pediatric injury prevention, clinical practice, and policy development. Interventions addressing behavioral risk factors, including helmet use and safe riding practices, should be reinforced through coordinated school- and community-based education initiatives. Clinical management may benefit from the identified high-severity injury patterns by prioritizing early detection and accurate triage of head, facial, and multisystem trauma in pediatric patients. Policy efforts should advance child-centered road safety by strengthening the implementation and enforcement of age-specific traffic regulations, expanding protected cycling infrastructure, and integrating environmental safety considerations into urban planning. Translating the configurational pathways revealed by the fsQCA into targeted public health and policy actions can meaningfully reduce the incidence and severity of pediatric e-bike injuries, while future investigations should assess the effectiveness of these interventions across diverse socio-economic and geographic settings to enhance their broader impact.

However, several limitations warrant acknowledgment. First, 22 studies included in this analysis were unevenly distributed geographically, with the majority originating from high-income, developed countries. This uneven distribution may influence the generalizability of our findings, as pediatric e-bike injury patterns, exposure, and healthcare access differ substantially across regions. In lower- and middle-income countries, differences in infrastructure, traffic regulations, helmet use, and emergency medical services may lead to distinct injury mechanisms and severity profiles. Additionally, socio-economic factors such as family income, parental supervision, and access to safety education can modulate both the likelihood and severity of injuries.

Second, although fsQCA allows the exploration of complex causal patterns across heterogeneous studies, the limited number of empirical cases constrains generalizability. Future work should expand the evidence base through systematic data harmonization and cross-country case inclusion to capture broader contextual diversity.

Third, some included studies lacked detailed reporting on exposure measures and behavioral variables, which may have influenced configurational calibration. Developing standardized reporting guidelines for e-bike injury studies could improve analytical precision.

Fourth, the fsQCA method identifies configurations of association but does not imply causal directionality. The mixed-method triangulation and prospective cohort designs could help validate these causal pathways.

## 6 Conclusion

This configurational analysis revealed that pediatric e-bike injury severity emerges from complex interactions among research quality, clinical measurement approaches, population characteristics, and behavioral contexts, rather than from isolated risk factors. Only through coordinated interventions spanning individual protection, device regulation, infrastructure investment, and behavioral guidance can we effectively reduce the burden of pediatric e-bike injuries while preserving the mobility benefits these devices offer.

## Data Availability

The original contributions presented in the study are included in the article/Supplementary material, further inquiries can be directed to the corresponding authors.
