# Supplementary material for: Configurational pathways to pediatric e-bike injury severity: an fsQCA study of global evidence
Source: Front Public Health. 2026 Jan 6;13:1738100. doi: 10.3389/fpubh.2025.1738100 (PMC12816185; doi:10.3389/fpubh.2025.1738100)
Supplement: Supplementary file 1 [file Table_1.DOCX]

***Supplementary Material***

**Table S1 Calibration of Condition Variables for fsQCA Analysis**

| **Condition Variable** | **Membership Score** | **Operational Definition / Criteria** | **Theoretical Correspondence** |
| --- | --- | --- | --- |
| **High Research Quality** | 1.0 | High-quality design (cohort or case-control), representative sample, confounder control, robust analysis | Public Health Systems Theory: reliable evidence generation |
|  | 0.67 | Moderate-quality design; some methodological limitations | Public Health Systems Theory |
|  | 0.33 | Low-quality design; major limitations in sample, confounders, or analysis | Public Health Systems Theory |
|  | 0.0 | Poor-quality design; non-representative, uncontrolled, weak analysis | Public Health Systems Theory |
| **Design Type** | 1.0 | Longitudinal (cohort) or case-control study | Complex Causality Theory: captures temporal and causal interactions |
|  | 0.0 | Cross-sectional study | Complex Causality Theory |
| **Quantitative Severity Indicators** | 1.0 | Standardized injury metrics (e.g., ISS, AIS) clearly reported | Clinical Epidemiology: systematic measurement of injury severity |
|  | 0.5 | Partial or non-standard severity metrics reported | Clinical Epidemiology |
|  | 0.0 | No quantitative severity indicators | Clinical Epidemiology |
| **Head/Facial Injury Focus** | 1.0 | Specific analysis of cranial or craniofacial trauma | Three-Element Model of Injury Epidemiology: host–agent–environment interaction |
|  | 0.67 | Descriptive mention of head/facial injury | Three-Element Model |
|  | 0.0 | Not addressed | Three-Element Model |
| **Multisystem or Lower Extremity Injury Focus** | 1.0 | Polytrauma or limb injuries included | Safety Ecology Model: ecological complexity of injury patterns |
|  | 0.0 | Absent | Safety Ecology Model |
| **Exclusively Pediatric Population** | 1.0 | Sample exclusively children/adolescents | Developmental & Public Health Theory: age-specific vulnerability |
|  | 0.5 | Mixed-age sample | Developmental & Public Health Theory |
|  | 0.0 | Adult-only sample | Developmental & Public Health Theory |
| **Behavioral and Environmental Risk Factor Analysis** | 1.0 | Includes helmet use, traffic exposure, or environmental hazards | Safety Ecology Model: child–environment–behavior interaction |
|  | 0.0 | Not included | Safety Ecology Model |

**Table S21 Character of Pediatric E-Bike Injury Studies**

| **Author** | **Year** | **Study Design** | **Objective** | **Injury Characteristics** | **Significant Results** |
| --- | --- | --- | --- | --- | --- |
| Avrahamovkraft et al. | 2022 | Observational Study | Compare injury patterns and severity between children riding e-bikes and classic bicycles | Injury Severity Score (ISS), head injuries, loss of consciousness, lower extremity injuries, orthopedic surgical interventions | Injury severity is significantly higher among e-bike patients compared to classic bicycle patients. |
| Bellity et al. | 2025 | Observational Study | Describe epidemiology of non-fatal injuries in children using electric and non-electric PMDs and identify risk factors for hospitalization | None specified | PMD use remains a major source of injury in children; more severe injuries occur among adolescents using electric PMDs. |
| Botton et al. | 2021 | Observational Study | Describe causes, injury types, and epidemiology of pediatric traffic accidents, comparing LEV to conventional bicycles and other vehicles | Head and neck injuries, lower extremity injuries, multi-system injuries, injury severity score | LEV accidents among children result in more severe injuries than other LNEVs. |
| Capua et al. | 2019 | Observational Study | Describe epidemiology and severity of pediatric e-bike injuries vs. manual bicycles | Head injuries, limb injuries, abdominal organ injuries, major trauma (ISS>9) | Pediatric e-bike injuries tend to be more severe than those sustained during manual bicycle riding. |
| Dimaggio et al. | 2020 | Observational Study | Compare injury risks of e-bikes, electric scooters, and pedal bicycles | Internal injuries, hospital admission, concussion, pedestrian collisions | E-bike and powered scooter use and injury patterns differ from traditional pedal bicycles. |
| Goodman et al. | 2023 | Observational Study | Compare injury rates among children on e-bikes, bicycles, and gasoline-powered bikes | Not specified | Highlights the importance of increasing e-bike safety and helmet use among children. |
| Gross et al. | 2018 | Observational Study | Characterize e-bike related injuries | Head injuries, facial injuries, fractures, lacerations, major trauma | E-bike injuries may involve serious trauma, with injury patterns resembling motorcycle injuries, particularly in children. |
| Hagel et al. | 2015 | Case-control Study | Investigate factors associated with severe bicycle injuries in youth | Severe injuries, male gender, helmet non-use, motor vehicle collision, paved surface, utilitarian riding | Bicycle-motor vehicle collisions increase the risk of severe injury in youth; adolescents are frequently affected. |
| Hermon et al. | 2020 | Observational Study | Report e-bike related injuries in children at a trauma center | Head injuries, upper limb injuries, lower limb injuries | E-bike related injuries in children are common, indicating a need for regulatory safety enhancement. |
| Hirsch et al. | 2024 | Observational Study | Determine impact of adding electric motors to RCs on pediatric injury severity and craniofacial fractures | Facial fractures, severe injuries, skull fractures | Addition of electric motors significantly increases risk of craniofacial fractures and severe injury in children. |
| Holland et al. | 2017 | Observational Study | Examine prevalence and impact of pediatric trauma and injuries on mortality and morbidity | Head and facial injuries, urethral trauma, death, drowning, corrosive injury, foreign body aspiration | Pediatric trauma remains prevalent; occurrence and severity of head and facial injuries are reduced by helmet legislation. |
| Huang et al. | 2023 | Observational Study | Assess status and epidemiology of road traffic injuries among e-bike riders | Road traffic injuries (RTIs), e-bike traffic injuries (ERTIs), risk behaviors (e.g., infrequent helmet use, riding on sidewalks, listening to music, etc.) | ERTIs prevalence among e-bike riders is 4.81%, with children under 16 most affected (9.84%). |
| James et al. | 2023 | Cohort Study | Describe characteristics and outcomes of major trauma related to electric scooters | Severe traumatic brain injury | Significant increase in scooter-related trauma over four years; higher proportion of severe TBI compared to bikes/motorcycles. |
| Lee et al. | 2024 | Observational Study | Investigate traumatic injury patterns associated with stand-up e-scooters versus bicycles | Crown fractures, tooth avulsion, crown-root fractures, tooth instability, extrusive luxation, root fractures, maxillofacial fractures | Stand-up electric scooters are associated with worse prognosis for maxillofacial trauma than bicycles. |
| Moati et al. | 2025 | Cohort Study | Describe incidence, severity, and secular trends of e-bike and e-scooter injuries in pediatric patients | Not specified | E-bike and e-scooter injuries in children are increasing in incidence and severity. |
| Teisch et al. | 2015 | Observational Study | Identify major injury patterns and outcomes in pediatric bicycle accidents | Isolated head, abdominal, or limb injuries; multiple injuries; abdominal, orthopedic, vascular surgery | Pediatric bicycle accidents more commonly occur in males aged 10-14 years. |
| Todorov et al. | 2024 | Observational Study | Analyze epidemiology, causes, and trends of pediatric brain and spinal injuries in Bulgaria | Concussion, spinal nerve root contusion, head trauma, skull fracture, hydrocephalus, thoracic spinal nerve contusion, vertebral fracture, brain tumor | Significant burden of pediatric traumatic brain and spinal injuries. |
| Trichinopoly Krishna et al. | 2021 | Observational Study | Assess EAPC-related safety issues and adequacy of EU/UK legislation to reduce injury risk | Head injuries | EAPCs associated with increased severity of head injuries; current helmet legislation may require revision. |
| Zhang et al. | 2018 | Observational Study | Identify prevalence and risk factors for e-bike/motorcycle-related road traffic injuries | Self-reported confusion, accident history, red-light running, child/adult passengers, riding on motorways, riding against traffic, traffic violations, inattentive riding, speeding, wrong lane riding | E-bike/motorcycle related road traffic injuries require enhanced safety interventions. |
| Zhao et al. | 2023 | Observational Study | Describe epidemiology and prognosis of pediatric electrical flash burns from e-bikes | Electrical burn injuries | Electrical flash burns from e-bike batteries are increasingly common in children. |
| Zhou et al. | 2024 | Observational Study | Propose priority measures to reduce e-bike related accidents and casualties | Not specified | Countermeasures are needed to improve e-bike safety. |
| Zmora et al. | 2019 | Observational Study | Analyze epidemiology, injury characteristics, and hospital resource use for pediatric e-bike injuries | Age, proportion of brain injury, CT scan usage, length of hospital stay, injury severity score, number of injuries per patient | E-bikes are a significant cause of severe injury in children, especially older children, compared to most traffic injuries. |

LEV: Light Electric Vehicle

LNEV: Light Non-Electric Vehicle

PMD: Personal Mobility Device

EAPC: Electrically Assisted Pedal Cycle

RC: Ride-on Cycle

ISS: Injury Severity Score

LOS: Length of Stay

TBI: Traumatic Brain Injury


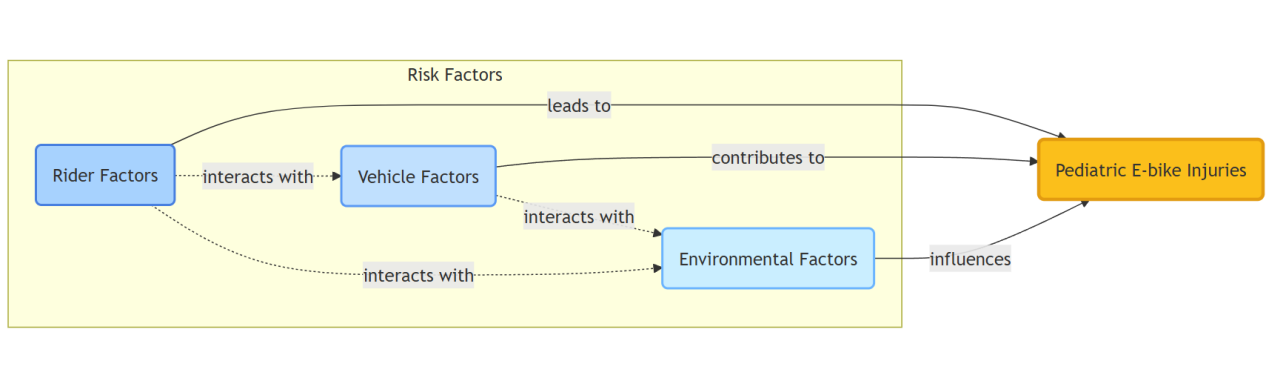


Figure S1 Types and Mechanisms of Pediatric Electric Vehicle Injuries.


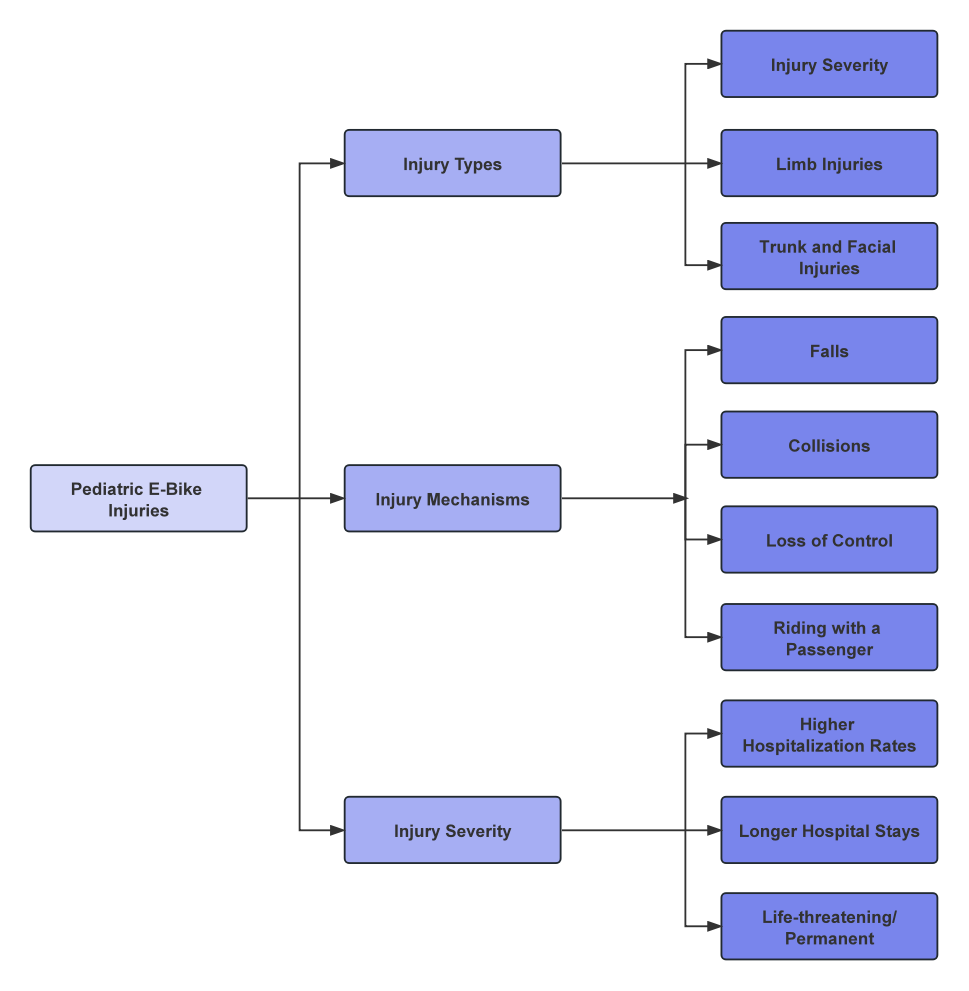


Figure S2 Multi-factorial Risk Pathways for Pediatric E-bike Injuries.
